# Towards a global partnership model in interprofessional education for cross-sector problem-solving

**DOI:** 10.1186/s12909-023-04290-5

**Published:** 2023-06-20

**Authors:** Fraide Ganotice, Binbin Zheng, Pauline Yeung Ng, Siu Chung Leung, Elizabeth Ann Barrett, Hoi Yan Celia Chan, Chad W. N. Chan, Kit Wa Sherry Chan, Linda Chan, M. K. Karen Chan, Siu Ling Polly Chan, So Ching Sarah Chan, Esther W. Y. Chan, Julie Chen, Yuet Ying Jessica Cheuk, Yin Kei Doris Chong, Yin Man Amy Chow, Kwok Pui Jody Chu, Hon Yin Brian Chung, Shun Yee Amy Ho, Julienne Jen, Jingwen Jin, Ui Soon Khoo, Ho Yan Angie Lam, May P. S. Lam, Suk Fun Veronica Lam, Pamela Pui-Wah Lee, Jetty Chung-Yung Lee, Chung Yin Feona Leung, Anna K. Y. Leung, Xiang Lin, Rebecca K. W. Liu, Wei Qun Vivian Lou, Pauline Luk, Lai Han Zoe Ng, Yee Man Alina Ng, Tin Wai Terry Ng, Lok Man Mary See, Jiangang Shen, Xiaoai Shen, Grace Szeto, Eliza Y. T. Tam, Kelvin Kai-Wang To, Wan-Yee Winnie Tso, Dana Vackova, Ning Wang, Runjia Wang, Hoi Yan Gloria Wong, K. T. Janet Wong, M. Y. Anita Wong, Yuen Ha Janet Wong, Kwan Yuk Jacqueline Yuen, Wai Yee Grace Yuen, Mine Orlu, George L. Tipoe

**Affiliations:** 1 Bau Institute of Medical and Health Sciences Education, The University of Hong Kong, Hong Kong, Hong Kong; 2 Critical Care Medicine Unit, the University of Hong Kong, Hong Kong, Hong Kong; 3 Emergency Medicine Unit, The University of Hong Kong, Hong Kong, Hong Kong; 4 Faculty of Education, The University of Hong Kong, Hong Kong, Hong Kong; 5 Department of Social Work and Social Administration, The University of Hong Kong, Hong Kong, Hong Kong; 6 School of Nursing and Health Studies, Hong Kong Metropolitan University, Hong Kong, Hong Kong; 7 Department of Psychiatry, The University of Hong Kong, Hong Kong, Hong Kong; 8 Department of Family Medicine and Primary Care, The University of Hong Kong, Hong Kong, Hong Kong; 9 Department of Family Medicine and Primary Care, The University of Hong Kong – Shenzhen Hospital, Shenzhen, China; 10 School of Nursing, The University of Hong Kong, Hong Kong, Hong Kong; 11 Department of Pharmacology and Pharmacy, The University of Hong Kong, Hong Kong, Hong Kong; 12 Department of Paediatrics and Adolescent Medicine, The University of Hong Kong, Hong Kong, Hong Kong; 13 Department of Professional Legal Education, The University of Hong Kong, Hong Kong, Hong Kong; 14 Department of Psychology, The University of Hong Kong, Hong Kong, Hong Kong; 15 Department of Pathology, The University of Hong Kong, Hong Kong, Hong Kong; 16 Faculty of Science, The University of Hong Kong, Hong Kong, Hong Kong; 17 School of Chinese Medicine, The University of Hong Kong, Hong Kong, Hong Kong; 18 UCL School of Pharmacy, University College London, London, UK; 19 School of Medical and Health Sciences, Tung Wah College, Hong Kong, Hong Kong; 20 Department of Microbiology, The University of Hong Kong, Hong Kong, Hong Kong; 21 School of Public Health, The University of Hong Kong, Hong Kong, Hong Kong

**Keywords:** Partnership model, Interprofessional education, Social interaction anxiety

## Abstract

**Objectives:**

A partnership model in interprofessional education (IPE) is important in promoting a sense of global citizenship while preparing students for cross-sector problem-solving. However, the literature remains scant in providing useful guidance for the development of an IPE programme co-implemented by external partners. In this pioneering study, we describe the processes of forging global partnerships in co-implementing IPE and evaluate the programme in light of the preliminary data available.

**Methods:**

This study is generally quantitative. We collected data from a total of 747 health and social care students from four higher education institutions. We utilized a descriptive narrative format and a quantitative design to present our experiences of running IPE with external partners and performed independent t-tests and analysis of variance to examine pretest and posttest mean differences in students’ data.

**Results:**

We identified factors in establishing a cross-institutional IPE programme. These factors include complementarity of expertise, mutual benefits, internet connectivity, interactivity of design, and time difference. We found significant pretest–posttest differences in students’ readiness for interprofessional learning (teamwork and collaboration, positive professional identity, roles, and responsibilities). We also found a significant decrease in students’ social interaction anxiety after the IPE simulation.

**Conclusions:**

The narrative of our experiences described in this manuscript could be considered by higher education institutions seeking to forge meaningful external partnerships in their effort to establish interprofessional global health education.

**Supplementary Information:**

The online version contains supplementary material available at 10.1186/s12909-023-04290-5.

## Key messages

- Factors in establishing an effective IPE external partnership model include complementarity of expertise, mutual benefits, internet connectivity and accessibility, and interactivity of design.
- Students ‘readiness for interprofessional learning (e.g., teamwork and collaboration, positive professional identity, roles and responsibilities) improved after the IPE programme.
- Social interaction anxiety significantly decreased after the IPE intervention (explicit priming) was implemented to support the development of smooth social interaction among students from different institutions.

## Introduction

The ability to work effectively as a member of interprofessional teams has been recognized as both a practice standard for different professions and a desirable graduate attribute of most universities [1, 2]. In healthcare, interprofessional collaboration is linked to optimal patient-centered care because, in contrast to the in-silo model, it leverages a team’s concerted expertise in managing the growing complexity of patient needs. Fostered through interprofessional education (IPE), an important assumption is that when professionals work in alliance, new practice-transforming solutions will emerge, medical errors will decline, and patient outcomes will improve [3].

Historically, IPE has been promoted with the goal of breaking down disciplinary silos, by providing healthcare students or professionals from two or more professions the opportunity to learn *about*, *from,* and *with*each other to optimize healthcare [4]. IPE is conventionally implemented as a cross-faculty programme that allows complementary disciplines within a university to work together in transforming the workplace. While within-University IPE is the default standard in many higher education institutions (HEIs; e.g., the study of El Ansari et al. [5]), the inherent limitation of this model is its inability to foster the development of students’ global and intercultural perspectives in health care. These desirable perspectives cannot be achieved within the bounds of a single institution, but need to be cultivated through external partnerships between universities. It is necessary to recognize that the inherently different perspectives of team members reflect the curricular and cultural influences imparted by the HEIs where they were trained. For this reason, a global IPE model which is co-created and co-implemented through the strategic cooperation or partnership of HEIs becomes relevant.

The Interprofessional Education and Collaborative Practice (IPECP) at the University of Hong Kong is one of the biggest interprofessional simulation programmes in Asia, training an annual average of 1,644 health and social care students. IPECP is an authentic experiential learning programme that aims to develop interprofessional collaboration-related competencies (e.g., values/ethics for interprofessional practice, roles/responsibilities for collaborative practice, interprofessional communication, interprofessional teamwork, and team-based care) among health and social care students [6], in response to the call of various health organizations to promote team-based healthcare [4, 7]. In the seventh year since its inception in 2016, and amidst the unprecedented changes to the landscape of education due to the COVID-19 pandemic, we reappraised and redesigned the IPECP programme to address both the students’ changing needs and the needs of the evolving healthcare delivery ecosystem. This provided the impetus for internationalization [8] as a way to evolve the programme.

Internationalization focusing on digital teaching channels is framed as a means to foster international cooperation, intercultural understanding, and a sense of global citizenship [9] early in students without the need to meet face-to-face. This provided an opportunity for four HEIs in Hong Kong and the United Kingdom (The University of Hong Kong, Tung Wah College, Hong Kong Metropolitan University, and University College London) to forge an IPE partnership (Table 1). In this international cross- and inter-institutional collaboration project, we set out to model how to advance IPE by co-designing and co-implementing creative IPE learning experiences notwithstanding the pandemic.

**Table 1:** Participating faculty and program

Faculty	Program
Li Ka Shing Faculty of Medicine, The University of Hong Kong (HKU)	1. Chinese Medicine
	2. Medicine
	3. Nursing
	4. Pharmacy and Pharmacology
Faculty of Science, HKU	5. Foods and Nutritional Sciences
Faculty of Education, HKU	6. Speech and Hearing Sciences
Faculty of Law, HKU	7. Law
Faculty of Social Sciences, HKU	8. Clinical Psychology (graduate level)
	9. Social Work and Social Administration (undergraduate level)
	10. Social Work and Social Administration (graduate level)
School of Medical Health and Sciences, Tung Wah College (TWC)	11. Physiotherapy
School of Nursing and Health Studies, Hong Kong Metropolitan University (HKMU)	12. Physiotherapy
UCL Faculty of Life Sciences, University College London School of Pharmacy	13. Pharmacy

Since its formal launch in 2016 [10], interprofessional education team-based learning (IPTBL) has been delivered using a blended learning approach that leverages on combined strengths of online learning and classroom face-to-face learning [11]. Although synchronous, face-to-face interaction is ideal if the goal is to simulate face-to-face teamwork. Owing to the differential time schedules of the 13 programmes involved from eight faculties (Table 1), the use of blended learning affords time and place flexibility to participating students and content experts from four HEIs to come together to learn *with*, *about*, and *from* one another.

In the course of developing the programme, we targeted an intervention that could yield desirable interprofessional collaboration competencies and facilitate smooth social interaction amongst students from different participating HEIs. We expected students’ potential interaction anxiety which might affect their engagement and achievement in learning progress [12, 13]. Acknowledging the importance of students’ smooth social interactions in spite of their diverse academic backgrounds and culture, in the present study, we initiated a simple experiment aimed at helping students who may be showing anxiety in social interactions in a culturally diverse IPE learning environment. We conducted a sentence-completion intervention and examined how this could reduce students’ interaction problems [14]. In the inclusion of this experiment, we hope to demonstrate the importance of designing a learning environment where students, regardless of their level of ease of social interaction, were supported.

After launching the IPE global partnership model using an online platform, the next important step for IPE was to conduct an initial clarificative evaluation [15] by revisiting the programme activities more closely to ensure their alignment with programme goals and outcomes. While the HKU, UCL, TWC, and HKMU partnership was established primarily to co-train our students with interprofessional learning competencies, we took advantage of this initiative to generate new research directions. To set the momentum for research partnership, we developed a research framework that outlined the goals and priorities and evoked the cooperation of specialists with diverse expertise from the involved HEIs. As a starting point, we aimed to understand if the global IPE model in a digital online format would yield desirable collaboration-related outcomes (e.g., teamwork and collaboration, professional identity, roles and responsibilities) similar to conventional face-to-face [16].

### The IPECP three-tier model

The IPE programme is a spiral model composed of three tiers (Tier 1: IPE literacy, Tier 2: IPE simulation, Tier 3: IPE collaborative practice). Tiers 1 and 2 were implemented through an online learning management system (LMS) called Open edX, which was founded by Harvard and Massachusetts Institute of Technology to cater to the needs of online collaborative learning [17]. We also used Zoom, Miro, Padlet, Metaverse, and Qualtrics embedded in Open edX. These were integrated into the “Asynchronous and Synchronous Interprofessional Education” to train health and social care students for collaborative practice. This model was based on a constructivist approach [18], in which activities were designed to provide students with experiential learning. We identified learning targets that were mapped alongside the Canadian National Interprofessional Education Competency Framework: 1. patient/client centeredness, 2. collaborative communication, 3. role understanding, 4. team functioning, 5. shared leadership and collaborative decision making, and 6. conflict resolution [19]; and Core Competencies for Interprofessional Collaborative Practice: 1. values and ethics, 2. roles and responsibilities for collaborative practice, 3. interprofessional communication, and 4. teamwork and team-based care [20].

**Fig. 1:**
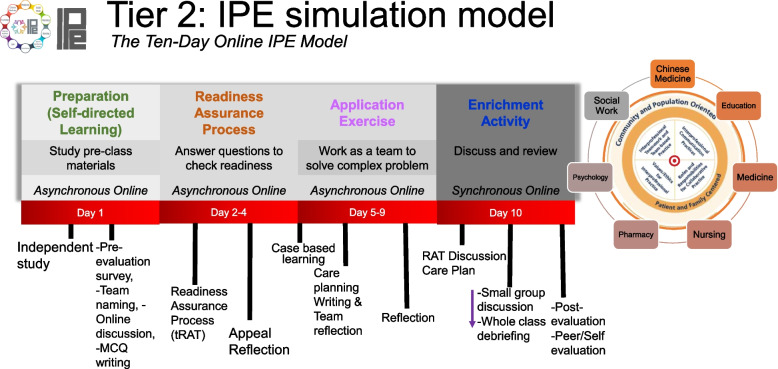
The Online IPECP Model

The “IPE Simulation” (Tier 2) was designed following a PRAE sequence of asynchronous (online) and synchronous activities (face-to-face). This acronym stands for a sequence of activities: Preparation, Readiness assurance, Application exercise, and Enrichment activity (Fig. 1). Consistent with the partnership model, clinical cases on Dementia and Fracture including all other learning activities were co-developed by all the content experts from the four HEIs through various discussions. These cases underwent a number of iterations to meet the suggestions of all the content experts involved and to ensure clarity, relevance, correctness, and cognitive load or appropriateness to the levels of the students. The activities were framed within Garrison et al.’s social constructivist framework called Community of Inquiry (CoI) [15] which highlights three essential elements of educational experience: social presence (encouraging connection with others), cognitive presence (meaning construction from learning experience), and teaching presence (activities surrounding the course design). This framework has been helpful for us in designing comprehensive IPE experiences to promote a community of inquiry in which meaningful learning experiences could be realized.

### The present study

In establishing partnerships with international and local HEIs, we considered a number of factors before launching the idea and before signing the letters of understanding (Table 2). These factors were taken into account as we sought to develop meaningful partnerships in an effort to provide our students with a relevant interprofessional global health education programme. To fine-tune the IPE programme, increase the likelihood that its implementation leads to desired outcomes, and provide a basis for monitoring and eventual impact evaluation, it is important to examine the effectiveness of this global partnership IPE model. In this connection, the aims of this study were threefold:

1. Describe the core components of the IPECP programme model,
2. Evaluate its effectiveness using the following indicators:
3. students’ behavioral change across the indices of interprofessional collaboration, including teamwork and collaboration, positive and negative professional identity, and roles and responsibilities; and
4. programme’s ability to facilitate social interaction adjustments, relatedness, and engagement in the IPE context;
5. Identify general programme areas needing improvement.

**Table 2:** The participants of this study (*N* = 747)

	Participants	Year level
*IPE Depression 2021*		
HKU MBBS	96 (33.7%)	Year 4
HKU Nursing	90 (31.6%)	Year 4
HKU Chinese Medicine	20 (7%)	Year 3
HKU Social Work	79 (27.7%)	Year 1 (Master)
Total	285 (100%) response rate: 91.34%	
*IPE Dementia 2022*		
HKU MBBS	40 (19.2%)	Year 4
HKU Nursing	83 (39.9%)	Year 4
UCL Pharmacy	8 (3.8%)	Year 3 (Master)
HKU Social Work	35 (16.8%)	Year 4 / Year 1 (Master)
HKU Speech and Hearing Science	42 (20.2%)	Year 5
Total	208 (100%); response rate: 54.73%	
*IPE Fracture 2022*		
HKU MBBS	41(16.2%)	Year 4
HKU Nursing	84 (33.1%)	Year 2
HKU Pharmacy	30 (11.8%)	Year 3
HKMU, TWC Physiotherapy	78 (30.7%)	Year 2/Year 3
HKU Social Work	21 (8.2%)	Year 1 (Master)
Total	254 (100%); response rate: 84.39%	

*HKU* The University of Hong Kong, *UCL* University College London, *HKMU* Hong Kong Metropolitan University, *TWC* Tung Wah College

We aim to contribute to the growing body of knowledge of IPE by modeling the importance of the global partnership IPE model in healthcare curricula. To our knowledge, no similar attempt has been undertaken to understand this global partnership IPE model; hence, this study is essential because it addresses this significant knowledge gap. We hope to build from the conversation on various IPE topics to represent the global IPE model we developed and describe all the parts to understand the psychological and pedagogical basis for which they are included in the model. This is an important step within which best practices in managing a global IPE partnership model may be uncovered. Additionally, we aim to model the cooperation and partnership of HEIs in providing a narrative of how HEIs can come together to provide students with a richer and more authentic learning experience.

## Methods

### Design

We utilized a descriptive narrative format in describing the programme and used a quantitative design to understand students’ potential gains in IPECP. To provide preliminary evidence to suggest the acceptability of the programme, the programme implementers and content experts used debriefing as a strategy for learning about and making future improvements. We conducted this investigation in pre-clinical IPE simulations: The Online IPECP Model (Fig. 1) consisted of around two hours of pre-class preparation and 3.5 h of the face-to-face session.

### Participants

We collected data from a total of 747 health and social care students with a mean age of 22.42 in the academic years 2020–2021 (*n* = 285) and 2021–2022 (*n* = 462) (Table 2). These students participated in any of the IPE simulations (Tier 2, explained in the results section) as part of their curricula. Recruitment of participants was facilitated by content experts of each of the participating HEIs. Students’ participation was completely voluntary, and we explained that their participation in the study would not affect their course grades. Participants signed the consent form to indicate their participation in the investigation. The content experts attended the debriefing which led to the identification of factors we considered in forging a partnership model in IPE.

### Measures and analysis

#### Readiness of students towards IPE

To estimate the readiness of students to engage in Online IPE, the Readiness for Interprofessional Learning Scale was administered before and after an IPE simulation intervention [21]. The 19 items were rated on a scale from 1 (strongly disagree) to 5 (strongly agree) under the following domains: teamwork and collaboration (9 items, “*Learning with other students will help me become a more effective member of a health care team*”; α = 0.94), negative professional identity (3 items, “*I don’t want to waste my time learning with other health-care students*”; α = 0.90), positive professional identity (4 items, “*Shared learning will help to clarify the nature of patient problems*”; α = 0.89), and roles and responsibilities (3 items, “*I’m not sure what my professional role will be*”; α = 0.83). This scale has been validated in Hong Kong students [22]. We reported here the Cronbach’s alpha reliability (α) based on the current data.

#### Behavior engagement and disaffection

To measure students’ engagement and disaffection, we used the two subscales of Engagement Versus Disaffection with Learning: Student Report in the IPE context: behavior engagement (5 items, “*In IPE, I work as hard as I can*; α = 0.92”), and behavior disaffection (5 items, “*When I’m in IPE, I just act like I’m workin*g; α = 0.84”) [23]. Responses are scaled from 0 (not at all true for me) to 3 (very true for me). This scale was administered after the Ten-Day Asynchronous and Synchronous Interprofessional Education. This scale was previously validated in IPE in the current setting [22].

#### Sense of relatedness

Aiming to understand the sense of relatedness of the students [24], we used four key items from the previous study. Items were rated from 1 (strongly disagree) to 4 (strongly agree). To understand students' sense of relatedness in two learning contexts e.g., peer and IPE, we adapted the original questionnaire measuring peer interaction (e.g., “*When I’m with peers in my discipline, I feel ignored*”; α = 0.73) to the IPE context (e.g., “*When I’m in IPE, I feel ignored*”; α = 0.85).

*Social Interaction Anxiety Scale (SIAS) and Social Phobia Scale*(SPS) [25]. The SIAS-6 measures general anxiety in terms of initiation and maintenance of social interactions. The SPS-6 intends to measure the experience of anxiety in the performance of various tasks while being examined by others. The items were rated from 0 (not at all characteristic or true of me) to 4 (extremely characteristic or true of me). These scales have been validated in HEIs [25].

We used analysis of variance (ANOVA) to understand potential differences in students’ behavioral engagement, behavioral disaffection, sense of relatedness in IPE, and sense of relatedness with peers. We used paired sample t-tests to compare the pretest and posttest scores on readiness for interprofessional learning, potential social interaction anxiety, and social phobia. For all the data analysis, we used the Statistical Package for Social Sciences (SPSS) Version 23.

## Results

As shown in Table3, we found significant differences between pre-and post-test scores in three indices of readiness for interprofessional learning: teamwork and collaboration, *t* (182) = -4.37, *p* < 0.01, positive professional identity, *t*(182) = -2.41, *p* < 0.025, roles and responsibilities *t*(182) = -2.77, *p* < 0.025.

**Table 3:** Mean comparison of students’ readiness for interprofessional learning (*n* = 183, IPE Depression, 2021)

RIPLS Dimensions	Pre-test	Post-test	*t*	*p*
	Mean (SD)	Mean (SD)		
Teamwork and Collaboration	3.72 (.77)	3.95 (.59)	-4.37	.00***
Negative Professional Identity	2.32 (.93)	2.39 (.97)	-.88	.38 ns
Positive Professional Identity	3.67 (.81)	3.81 (.61)	-2.41	.02*
Roles and Responsibilities	2.79 (.75)	2.96 (.69)	-2.77	.01**

Teamwork—interaction of two or more individuals who interdependently work for a common purpose; professional identity—a sense of oneself reflecting the attitudes, values, and knowledge specific to a professional group; roles and responsibilities—refers to one's position on a team including related tasks and duties he tasks and duties of their particular role or job description. 1 = strongly disagree—5 = strongly agree; *p* = *** < .001; ** *p* < .01; * *p* < .05; ns = not significant

**Fig. 2:**
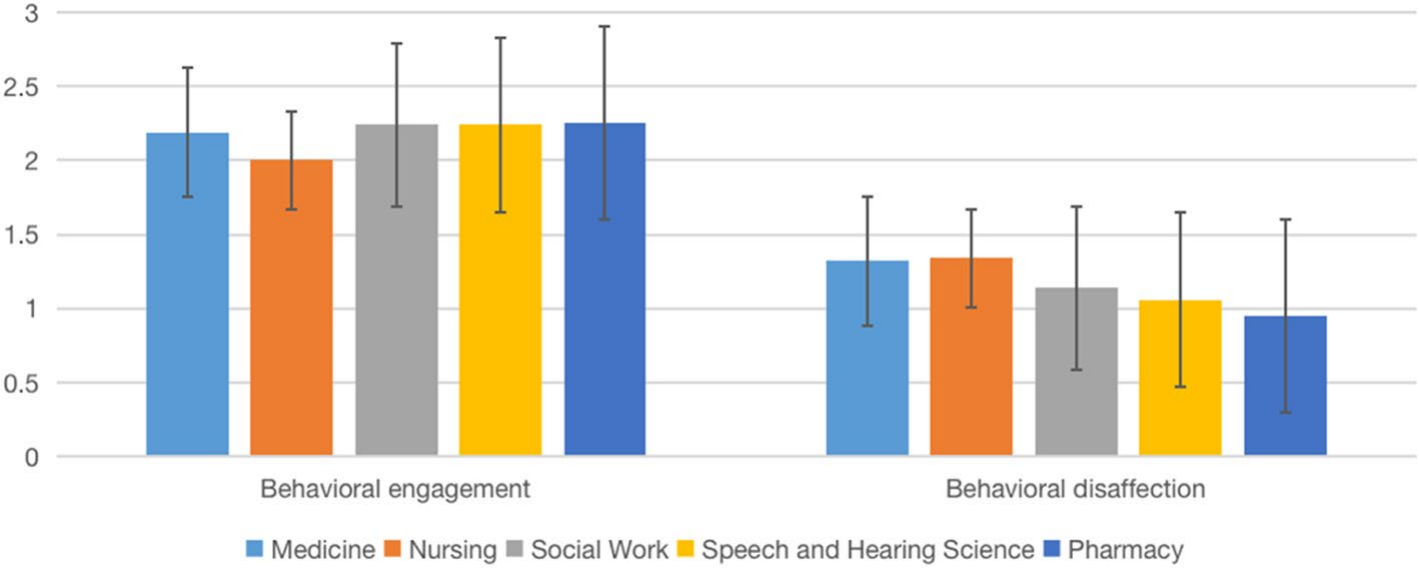
Comparison of means among disciplines on engagement (IPE Dementia, 2022)

**Fig. 3:**
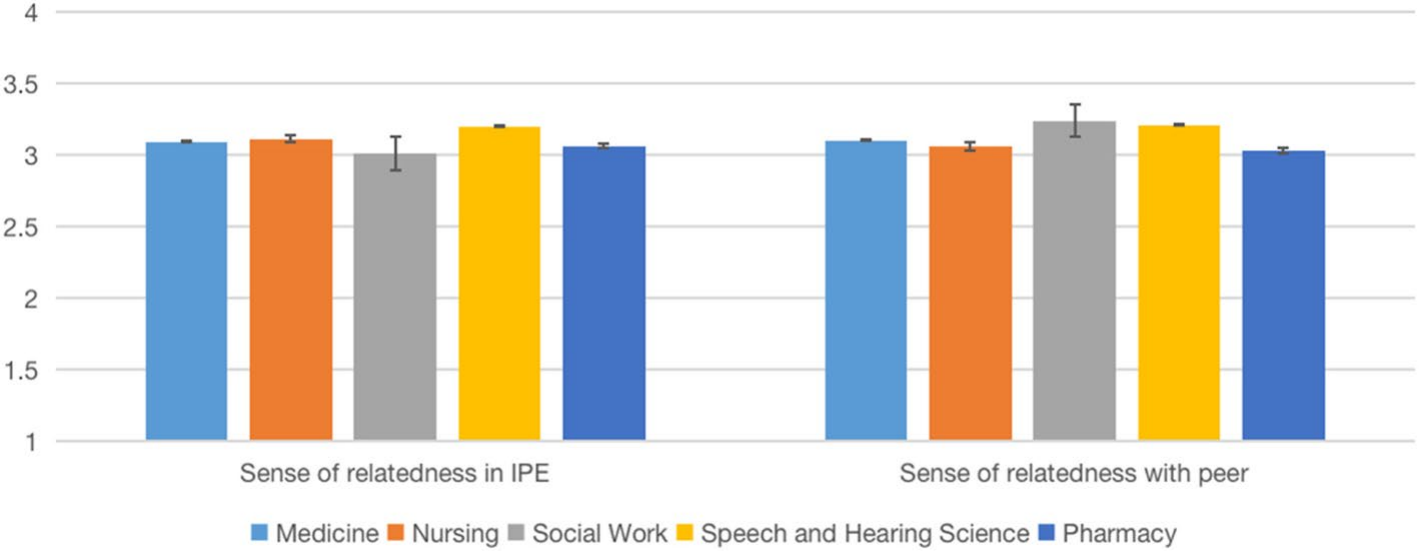
Comparison of means among disciplines on relatedness (IPE Dementia, 2022)

**Fig. 4:**
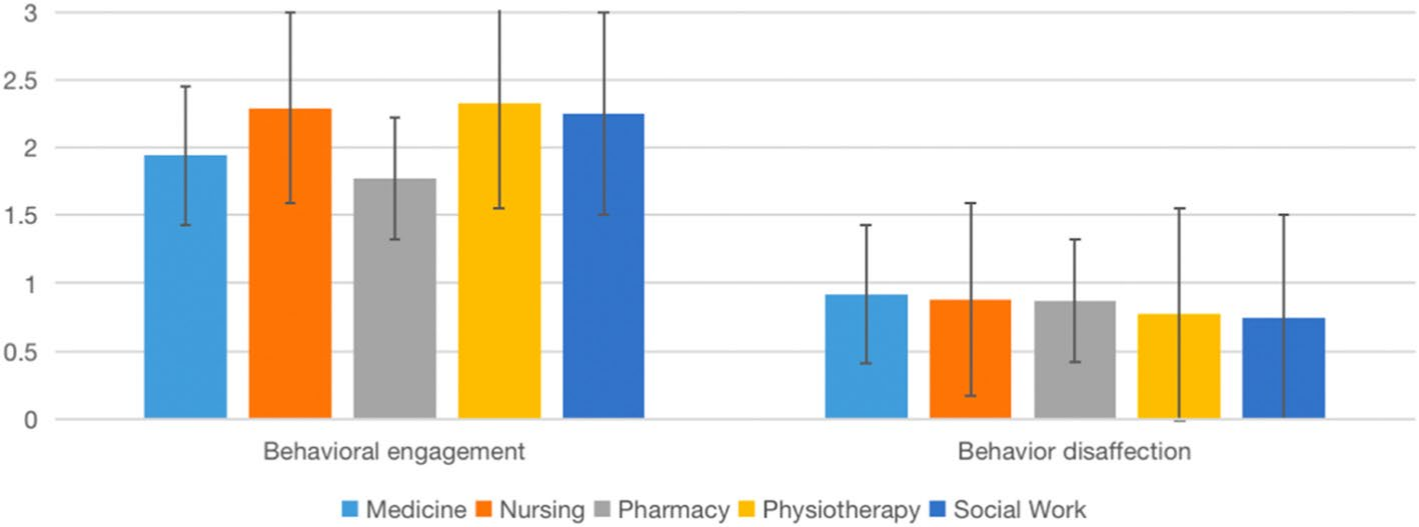
Comparison of means among disciplines on engagement (IPE Fracture, 2022)

**Fig. 5:**
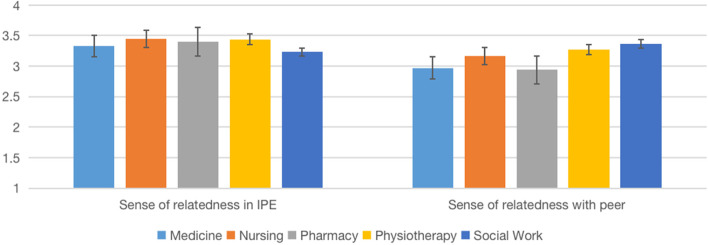
Comparison of means among disciplines on relatedness (IPE Fracture, 2022)

We examined potential differences between disciplines in behavioral engagement and disaffection and sense of relatedness on the post-test data (Table 4, Figs. 2, 3, 4, 5). One-way ANOVA results showed no significant discipline effect in behavioral engagement (*F*(4,196) = 2.10, *p* = 0.083), behavioral disaffection (*F*(4,196) = 2.08, *p* = 0.085), sense of relatedness in IPE (*F*(4,201) = 1.13, *p* = 0.346) and sense of relatedness with peers (*F*(4,201) = 1.50, *p* = 0.203).

**Table 4:** Comparison of means among disciplines on engagement, disaffection, and relatedness (IPE Fracture, 2022)

Variables	Mean (*SD*)					*F*	*p*	Post hoc comparison
	Medicine (*n* = 26)	Nursing (*n* = 75)	Pharmacy (*n* = 19)	Physiotherapy (*n* = 33)	Social Work (*n* = 14)			
Behavioral engagement	1.94 (.60)	2.29 (.48)	1.77 (.44)	2.33 (.51)	2.25 (.56)	6.12	.000	(Med < Nurs, Physio; Phar < Nurs, Physio
Behavior disaffection	.92 (.73)	.88 (.56)	.87 (.48)	.77 (.64)	.75 (.67)	.357	.839	ns
Sense of relatedness in IPE	3.33 (.57)	3.45 (.49)	3.40 (.51)	3.44 (.63)	3.23 (.94)	.654	.625	ns
Sense of relatedness with peer	2.97 (.51)	3.17 (.50)	2.94 (.74)	3.27 (.45)	3.37 (.56)	2.536	.042	ns

Behavior engagement and disaffection: 0 (not at all true)—3 (very true); Sense of relatedness: 1(not at all true)—4 (very true); *ns* Not significant

We performed a similar analysis with data from the IPE Fracture module (Table 4). One-way ANOVA results showed a significant discipline effect in behavior engagement (*F*(4,162) = 6.125, *p* = 0.000), no significant effect in behavior disaffection (*F*(4,160) = 0.357, *p* = 0.839), and a sense of relatedness in IPE (*F*(4,162) = 0.654, *p* = 0.625), as well as the marginal effect on the sense of relatedness with a peer (*F*(4,162) = 2.536, *p* = 0.042).

We conducted a paired sample t-test to compare the pre-test and post-test scores on the Social Interaction Anxiety Scale (SIAS) to understand how IPE social interaction with sentence-completion priming can help reduce students’ potential interaction anxiety (Table 5). Results revealed a significant difference (*t* (20) = 1.724, *p* = 0.01) in the pre-test (M = 2.02, *SD* = 0.34) and the post-test (M = 1.70, *SD* = 0.86) in terms of the degree of students' Social Interaction Anxiety. A paired-sample t-test was also conducted to compare the score on the Social Phobia Scale (SPS) before and after the IPE activity. The mean of the pre-test results was 1.98 (*SD* = 0.28) compared to the mean of 1.40 (*SD* = 0.83) in the post-test, which was a significant difference (*t* (18) = 2.77, *p* = 0.01).

**Table 5:** Changes in levels of anxiety and phobia among participants (IPE Dementia, 2022)

Variable	Pre-test	Post-test	n	*t*
	Mean (*SD*)	Mean (*SD*)		
Social interaction anxiety	2.02 (0.34)	1.70 (0.86)	21	1.724*
Social Phobia	1.98 (0.28)	1.40 (0.83)	19	2.77**

The participants in this study were those who scored high in measures of social anxiety and social phobia administered at Time 1 – pre-test; *p < .1; **p < .05

The content experts, together with the programme coordinator, attended a debriefing where they discussed their experiences that led to the creation of a partnership model in IPE (Table 6).

**Table 6:** Factors considered in forging a global IPE collaboration

Institutional goals and priorities	Our consultation with HEIs aimed at a mutually beneficial partnership. We discussed how our cooperation was aligned with the achievement of our institution’s thrust and priorities
Complementarity of expertise	The appropriateness and combination of pool expertise or discipline in a team were of primary importance. In the initial identification of HEIs to join the program, we considered the expertise of the target HEI to complement and not duplicate the existing disciplines. For example, the inclusion of Physiotherapy students from Hong Kong Metropolitan University and Tung Wah College complements the existing disciplines
Internet connectivity and electronic platform	The Open edX learning management system adopted for IPECP has been finetuned over the years to especially meet the need of increasing numbers of students who concurrently use Open edX
Interactivity of design	The IPECP design is built primarily with various carefully designed and structured activities to facilitate learning in groups in line with the achievement of interprofessional collaborative competencies. Using Open EdX LMS, these activities were distributed into a sequence: 1. Preparation, 2. Readiness Assurance Process, 3. Application Exercise, 4. Enrichment Activity
The number of students	For a cross-institutional IPE, while we expect a huge number of complementary health and social care students, we aimed for a balanced number of complementary expertise to even out the number of expertise in a team. This is challenging given that students’ enrolment largely varies
Authenticity of learning experiences	In developing the simulation cases, content experts aimed for authenticity in depicting the real and common experiences of patients in all the simulation cases
Time differences	Small group team members simulating interprofessional healthcare teams need to identify a common time to collaborate online. We planned ahead and arranged a schedule that was most convenient for all participating HEIs
Positive relationships	Our collaboration was anchored on positive relationships. All the teaching and learning activities were duly approved by all the participating HEIs. We were also clear about the responsibilities of collaborating HEIs

## Discussion

The recognition that members of healthcare teams in workplace clinical settings usually obtained their pre-licensure training from different institutions of higher learning suggests the need to align this workplace reality with the IPE training in medical schools. This recognition leads to the need for an inter-institutional or global IPE.

To the best of our knowledge, this is the first study on IPE global partnership model with multiple local and international partners. The strengths of our study were the representation of study participants from 13 different health and social care programmes from four HEIs, the integration of our own experiences, and the students’ performance data using standardized and validated questionnaires. We believe that we were able to drive innovation in the way IPE is developed and implemented through modeling cross-institutional collaboration to benefit our students.

Our data suggest the acceptability of implementation outcomes [26] of an Online IPE jointly implemented by collaborating HEIs. Our experience in co-implementing the global IPE was a meaningful learning opportunity both for content experts and students. From our prior experience, we learned that an IPE programme that was previously hybrid in format and delivered for a sole university could be successfully co-implemented and offered completely online. The integration of interprofessional care planning underpinned by constructive controversy using the online platform MIRO board (was an important innovation designed to foster interprofessional teamwork and collaboration.

An interesting observation noted by the facilitators and content experts relates to the demonstration of students’ positive interdependence in social interactions during live synchronous activities. We observed that students showed higher motivation to do well in the context of mixed team membership from different institutions, in contrast with a team with members from a single institution. Students were very active in various team activities and were willing to turn on their cameras. This observation may be explained by the ability of social situations or associational forces to influence one’s tendency to do well, especially in social settings known as social facilitation [27]. In particular, it may be the case of co-action effects (tendency to do well as others are doing similar tasks) and audience effects (tendency to do well in front of an audience).

Our students in IPECP were diverse and dispersed, with an average of five disciplines in a single simulation model. Given the diversity of students’ backgrounds, we underscored the development of *a shared or collaborative mindset*, providing the team with a compelling direction to adopt a collective healthcare team [28]. We provided the teams with the opportunity to identify healthcare management goals explicitly. Based on our observation, despite their differential disciplinary expertise, the shared mindset which we emphasized in whole-class briefings provided them motivation and direction. We reiterated the core value of interdependence by demonstrating teamwork and collaboration. We emphasized to the students the need to understand their professional identity in the context of teams, the roles, responsibilities, and partnerships among various professionals. Mutual trust, respect, communications, and accountability are crucial elements for synergistic work outcomes.

We believe that our partnership with other HEIs is a strong starting point in which we can jointly promote the advancement of science and scholarship of IPE through research. We examined if Online IPE can yield desirable effects that are associated with face-to-face delivery. Similar to face-to-face IPE [29], our data suggest that the Online IPE model can develop students’ teamwork and collaboration, positive professional identity, and roles and responsibilities (Table 7). Additionally, our data in running IPE Dementia and Fracture simulations suggest that students, in general, yield high behavioral engagement (and low disaffection) and a sense of relatedness in IPE and peers (Figs. 2, 3, 4, 5). We would like to emphasize that the non-statistically significant programme effect was in line with our expectations, suggesting the effect of IPE across all the programmes. Aside from behavioral engagement, which was found to be significantly lower among medical and pharmacy students than nursing and physiotherapy students, there were no significant post-test differences in students' low behavioral disaffection and sense of relatedness, suggesting equal benefits among programmes. Taken together, these pieces of evidence suggest the effectiveness of the programme.

**Table 7:** Comparison of means among disciplines on engagement (IPE Dementia, 2022)

Variable	Mean (*SD*)					*F*	*p*	*η*^*2*^
	Medicine (*n* = 40)	Nursing (*n* = 82)	Social Work (*n* = 34)	Speech and Hearing Science (*n* = 42)	Pharmacy (*n* = 8)			
Behavioral engagement	2.19 (.11)	2.00 (.56)	2.24 (.49)	2.24 (.43)	2.25 (.78)	2.10	.083 ns	.041
Behavioral disaffection	1.32 (.86)	1.34 (.61)	1.14 (.55)	1.06 (.53)	0.95 (.50)	2.08	.085 ns	.041
Sense of relatedness in IPE	3.09 (.63)	3.11 (.47)	3.01 (.59)	3.20 (.44)	3.06 (.35)	1.13	.346 ns	.022
Sense of relatedness with peer	3.1 (.72)	3.06 (.49)	3.24 (.42)	3.21 (.30)	3.03 (.41)	1.50	.203 ns	.029

Behavior engagement and disaffection: 0 (not at all true)—3 (very true)

Sense of relatedness: 1(not at all true)—4 (very true); *ns* Not significant

We wish to emphasize that we planned ahead to mitigate potential students’ social interaction problems, given the mix of students from different expertise, faculties, and HEIs. To do this, we built from social psychology ideas [14] and conducted a simple experiment aimed at facilitating positive social interaction of students across HEIs, which explored interaction anxiety through explicit priming (sentence-completion test about IPE). Our data suggest that there was a significant decrease in social interaction anxiety and social phobia after the intervention by explicit priming, suggesting that the environment can be designed to help students overcome social interaction problems (Table 6).

### Programme challenges and opportunities for improvement

While global IPE is important for preparing health and social care students for future collaborative efforts, we experienced a number of difficulties in its implementation. We summarized these challenges and proposed actions in response to these limitations. The significant involvement of facilitators was one of the challenges we encountered. Given that IPECP is a large-scale inter-institutional collaboration, this necessitates a big number of facilitators. In line with this, we involved and trained near-peer-teachers (NPTs) as floating facilitators who rotated through teams. The time difference between involved HEIs was an additional challenge, suggesting the need to plan ahead to identify common times when students can meet. Monitoring team interactions was also a challenge. Many of the team activities were designed to be completed asynchronously. The use of learning analytics was necessary to ease the monitoring process of team progress in completing their tasks.

This study is not without limitations. *First*, in terms of participants, they were composed of only those who volunteered to participate in this study. Furthermore, there was a great difference in the number of participants from four HEIs who volunteered to participate in this investigation. *Second*, the self-report nature of the questionnaires was influenced by social desirability bias, although the anonymity of participants was ensured. Third, even if we have a large number of participants (*N* = 747), this number was not representative of the four HEIs. These limitations notwithstanding, we believe that these do not undermine the strength of this paper which is the integration of both descriptive and quantitative data collected from a large-scale global IPE model. While we know of various face-to-face IPE developed and implemented for a single institution [30, 31], the present study extends our understanding of the considerations in forging global IPE co-implemented by collaborating HEIs.

## Conclusion

We end by reflecting on our journey in co-developing global IPECP. With clear common goals shared by collaborating HEIs and institutional commitment, cross-institutional collaboration provides a win–win situation for all. The identified areas for improvement from our evaluation suggest that no collaboration is perfect. However, we are optimistic that no barrier is insurmountable with the synergy of our collective efforts to champion global IPE to revolutionize how care is delivered. We hope that our partnerships in developing Global IPE will serve as a model for school administrators to remain committed to designing innovative programmes to equip students with skills that will enable them to thrive in the twenty-first century workplace.

## Supplementary Information

**Additional file 1. **Appendixes.

## Data Availability

The dataset is available from the corresponding author on reasonable request.
