# Supplementary material for: Towards a global partnership model in interprofessional education for cross-sector problem-solving
Source: BMC Med Educ. 2023 Jun 20;23:457. doi: 10.1186/s12909-023-04290-5 (PMC10283208; doi:10.1186/s12909-023-04290-5)
Supplement: Supplementary file 1 — Additional file 1. Appendixes. [file 12909_2023_4290_MOESM1_ESM.docx]

**Appendixes**

**Validated Scale A: The Readiness for Interprofessional Learning Scale**

Instructions: Please indicate the degree to which you agree or disagree with the statement by drawing a circle around the number of the response that best expresses your feeling. The scale is as follows: 1 = strongly disagree, 2 = disagree, 3 = neutral, 4 = agree, 5 = strongly agree.

| 1. Learning with other students will help me become a more effective member of a health care team. | 1 | 2 | 3 | 4 | 5 |
| --- | --- | --- | --- | --- | --- |
| 1. Patients would ultimately benefit if health-care students worked together to solve patient problems. | 1 | 2 | 3 | 4 | 5 |
| 1. Shared learning with other health-care students will increase my ability to understand clinical problems. | 1 | 2 | 3 | 4 | 5 |
| 1. Learning with health-care students before qualification would improve relationships after qualification. | 1 | 2 | 3 | 4 | 5 |
| 1. Communication skills should be learned with other health-care students. | 1 | 2 | 3 | 4 | 5 |
| 1. Shared learning will help me to think positively about other professionals. | 1 | 2 | 3 | 4 | 5 |
| 1. For small group learning to work, students need to trust and respect each other. | 1 | 2 | 3 | 4 | 5 |
| 1. Team-working skills are essential for all health care students to learn. | 1 | 2 | 3 | 4 | 5 |
| 1. Shared learning will help me to understand my own limitations. | 1 | 2 | 3 | 4 | 5 |
| 1. I don’t want to waste my time learning with other health-care students. | 1 | 2 | 3 | 4 | 5 |
| 1. It is not necessary for undergraduate health-care students to learn together. | 1 | 2 | 3 | 4 | 5 |
| 1. Clinical problem-solving skills can only be learned with students from my own department. | 1 | 2 | 3 | 4 | 5 |
| 1. Shared learning with other health-care students will help me to communicate better with patients and other professionals. | 1 | 2 | 3 | 4 | 5 |
| 1. I would welcome the opportunity to work on small-group projects with other health-care students. | 1 | 2 | 3 | 4 | 5 |
| 1. Shared learning will help to clarify the nature of patient problems | 1 | 2 | 3 | 4 | 5 |
| 1. Shared learning before qualification will help me become a better team worker. | 1 | 2 | 3 | 4 | 5 |
| 1. The function of nurses and therapists is mainly to provide support for doctors. | 1 | 2 | 3 | 4 | 5 |
| 1. I’m not sure what my professional role will be. | 1 | 2 | 3 | 4 | 5 |
| 1. I have to acquire much more knowledge and skills than other health-care students. | 1 | 2 | 3 | 4 | 5 |

RIPLS Subscales:

1. Teamwork and collaboration (items 1 – 9)
2. Negative Prof Identity (items 10-12)
3. Positive Professional identity (items 13 – 16)
4. Roles and responsibility (items 17 – 19)

**Validated Scale B:  Engagement Versus Disaffection with Learning: Student Report**

Instructions: Please rate how you feel when participating IPE programme

(Response scale: 1-Not at all true; 2-Not very true; 3-Sort of true; 4-Very true)

Behavioral Engagement

| 1. I try hard to do well in IPE. | 1 | 2 | 3 | 4 |
| --- | --- | --- | --- | --- |
| 2.In IPE, I work as hard as I can. | 1 | 2 | 3 | 4 |
| 3.When I’m in IPE, I participate in class discussions. | 1 | 2 | 3 | 4 |
| 4.I pay attention in IPE. | 1 | 2 | 3 | 4 |
| 5.When I’m in IPE, I listen very carefully. | 1 | 2 | 3 | 4 |

Behavioral Disaffection

| 1.When I’m in IPE, I just act like I’m working. (–) | 1 | 2 | 3 | 4 |
| --- | --- | --- | --- | --- |
| 2.I don’t try very hard at IPE. (–) | 1 | 2 | 3 | 4 |
| 3.In IPE, I do just enough to get by. (–) | 1 | 2 | 3 | 4 |
| 4.When I’m in IPE, I think about other things. (–) | 1 | 2 | 3 | 4 |
| 5.When I’m in IPE, my mind wanders. (–) | 1 | 2 | 3 | 4 |

**Validated Scale C:  Social Interaction Anxiety Scale (SIAS-6) and Social Phobia Scale (SPS-6)**

Instructions: For each item, please tap the bx to indicate the degree to which you feel the statement is characteristic or true for you. Use the following scale: 0 – Not at all characteristic or true of me, 1 - Slightly characteristic or true of me, 2 - Moderately Characteristic or true of me, 3 – Very characteristic or true of me, 4 – Extremely characteristic or true of me

Social Interaction Anxiety

| 1. I have difficulty making eye contact with others | 1 | 2 | 3 | 4 | 5 |
| --- | --- | --- | --- | --- | --- |
| 2. I find it difficult mixing comfortably with the people I work with | 1 | 2 | 3 | 4 | 5 |
| 3. I tense up if I meet an acquaintance on the street | 1 | 2 | 3 | 4 | 5 |
| 4. I feel tense if I am alone with just one person | 1 | 2 | 3 | 4 | 5 |
| 5. I have difficulty talking with other people | 1 | 2 | 3 | 4 | 5 |
| 6. I find it difficult to disagree with another’s point of view | 1 | 2 | 3 | 4 | 5 |

Social Phobia

| 1. I get nervous that people are staring at me as I walk down the street | 1 | 2 | 3 | 4 | 5 |
| --- | --- | --- | --- | --- | --- |
| 2. I worry about shaking or trembling when I’m watched by other people | 1 | 2 | 3 | 4 | 5 |
| 3. I would get tense if I had to sit facing other people on a bus or train | 1 | 2 | 3 | 4 | 5 |
| 4. I worry I might do something to attract the attention of other people | 1 | 2 | 3 | 4 | 5 |
| 5. When in an elevator, I am tense if people look at me | 1 | 2 | 3 | 4 | 5 |
| 6. I can feel conspicuous standing in a line | 1 | 2 | 3 | 4 | 5 |
